# Presence of pepsin in laryngeal tissue and saliva in benign and malignant neoplasms

**DOI:** 10.1042/BSR20200216

**Published:** 2020-11-13

**Authors:** Željko Zubčić, Tihana Mendeš, Andrijana Včeva, Hrvoje Mihalj, Vjeran Bogović, Stjepan Grga Milanković

**Affiliations:** 1 Department of Otorhinolaryngology and Maxillofacial Surgery, Faculty of Medicine, Josip Juraj Strossmayer University of Osijek, J. Huttlera 4, Osijek 31 000, Croatia; 2 Department of Otorhinolaryngology, Head and Neck Surgery University Hospital Centre Osijek, J. Huttlera 4, Osijek 31 000, Croatia

**Keywords:** laryngeal neoplasms, laryngopharyngeal reflux, pepsin

## Abstract

**Objectives:** The current study was performed to determine the presence of pepsin in saliva and laryngeal tissue among participants with benign and malignant laryngeal neoplasms.

**Study design:** Case–control study included three groups of patients with: (1) benign laryngeal neoplasms, (2) malignant laryngeal neoplasms and (3) control subjects without symptoms or signs of laryngopharyngeal reflux (LPR).

**Methods:** Eighty-one voluntary participants were included into study. They were recruited from a group of patients with histologically proven benign and malignant laryngeal neoplasms and in case of control subjects among patients with nasal septum deformation without symptoms of LPR.

Morning saliva samples were collected preoperatively. Tumor biopsies were collected by directoscopy of larynx and the control samples from interarytenoid unit of larynx. All samples were analyzed by Enzyme-Linked Immunosorbent Assay (ELISA) and Immunohistochemistry.

**Results:** Pepsin was found in all samples of saliva and tissue biopsies in groups with malignant and benign neoplasms. The highest concentration of pepsin was found in a group of patients with malignant laryngeal neoplasms. Patients with benign laryngeal neoplasms had lower concentrations and the control subjects presented with the lowest concentration of pepsin measured from their saliva. Differences were not statistically significant. Immunohistochemical (IHC) analysis showed the largest number of high positive samples in the group of malignant lesions.

**Conclusion:** These results suggest that pepsin and LPR can contribute to the development of benign and malignant laryngeal neoplasms. Further prospective studies, with far more patients, are necessary to prove the role of pepsin in multifactorial etiology of laryngeal neoplasms.

## Introduction

Various tumors develop in the larynx due to different origins, various tissue histogenesis and mucosal features. Regarding their biological behavior, tumors are divided into benign and malignant, which has high clinical significance. The causes of tumor formation have not been established neither for benign, nor malignant neoplasms. Among the external risk factors, in the first place is permanent exposure of the mucosa and other tissue of the larynx to various mechanical, chemical, physical, inflammatory and functional irritations [1]. The latest hypothesis of etiology benign and malignant lesions in the larynx, as well as many other respiratory diseases, is based on laryngopharyngeal reflux (LPR) and pepsin activity as one of the possible factors which are causing these diseases. Despite many researches and publication of clinical reports, there is no general opinion on whether LPR is associated with benign and malignant lesions of the larynx. Still there are no established data for LPR as a definitive risk factor for benign and malignant lesions. Until recently, LPR was thought to be influencing epithelization and recurrence of laryngeal polyps or Reinke edema (RE) in vocal cords, after partial or total decortication [2]. New findings show that acidic environments compromise epithelial barrier function without gross structural changes [3]. Laryngeal tissues are essentially resistant to damage at pH 4.0, but are damaged when pepsin is present. This suggests that in LPR, pH 4.0 or above, refluxate would only be damaging if it contained pepsin [4]. Mucosa of the upper esophageal tract is more sensitive to gastric contents than the esophageal mucosa [5]. Primarily pH metric studies showed that these patients have LPR [6].

The laryngeal mucosa is extremely sensitive to the action of gastric contents (acid and non-acid reflux) [7–9]. Even in minimal biliary reflux, the non-acid reflux consists of pepsin and conjugated bile acids. Acid and non-acid reflux have a synergistic effect on the mucosa of the upper aerodigestive tract. Therefore, three reflux episodes per week in larynx may cause significant damage to the mucosal tissue of these organs [10]. Pepsin is an enzyme proteinase produced by parietal cells of the gastric mucosa in the inactive form, pepsinogen. The pepsinogen at the pH value 2 passes into the active form of pepsin. The maximum activity of pepsin is at pH 2 and is inactivated when the pH is above 6.5 [11]. Pepsin remains stable but inactive at pH values up to 8. Afterward, pepsin causes decomposition of carboanhydrate III (CAIII), an enzyme responsible for the formation of bicarbonate ions. Bicarbonate ions directly neutralize acidic gastric contents in the laryngeal mucosa. With deposition of CAIII, protective role of bicarbonate ions in the laryngeal mucosa is lost [12,13].

It is proven that pepsin inhibits expression and depletion of protective proteins such as mucin 2, mucin 3, mucin 5A, mucin 5B, sept 53, sept 70 and E-cadherin proteins that play an important role in maintaining cellular integrity of the epithelium. The lack of these proteins leads to a change in the cellular response to trauma and causes the damage to the mucosal membrane [14].

LPR is considered to be a significant risk factor for the development of planocellular carcinoma (PCC) but the causal relationship has not yet been proven. It is known that chronic return of gastric content to the esophagus can cause the metaplasia of the mucosa and consequently increase the risk of developing adenocarcinoma. Studies of biopsy culture cells of normal larynx cell and FaDo PCC hypopharyngeal cells by microRNA and Western blot analysis showed that pepsin added to cell cultures stimulates cell proliferation and significantly changes the expression of 27 genes linked to carcinogenesis and changes the expression of 22 microRNAs otherwise altered in the head and neck carcinomas. Pepsin was determined by Western blot analysis in 60% of laryngeal carcinoma biopsy and was not found in control samples [15].

Reflection on pepsin and LPR could change the current understanding of the etiopathogenesis of benign and malignant diseases of larynx, change preventive and therapeutic approaches and their effectiveness in diagnosis and treatment.

## Materials and methods

This case–control study involved 81 participants in the Otorhinolaryngology and Head and Neck Surgery Clinic Osijek during 3 years from the beginning of the research in 2015; 29 women and 52 men (ranging from 20 to 77 years). They were divided into three groups: (a) 31 (38%) subjects with benign lesions in the larynx (24–67 years, median 46.57 years), (b) 25 (31%) subjects with malignant lesions in the larynx (41–77 years, median 58.50) and (c) 25 (31%) control subjects without symptoms and signs of LPR (20–73 years, median 45.52 years).

In the first group, the only diagnosis was PCC 25, while laryngeal papillomatosis (PAP), vocal cord polyp (VP) 14 and RE 10 were most commonly diagnosed in the group with benign changes. In the group of participants with malignancies, seven subjects had stage T1, ten had T2 stage, five had T3 stage and three had stage T4.

The control group consisted of patients in the Otorhinolaryngology and Head and Neck Surgery Clinic, who were hospitalized for surgical treatment of the nasal septum deviation and did not have symptoms and signs of LPR. During the screening of the control participants, the Belafski questionnaire was used (Reflux finding scores, Reflux symptom index), which was enough to suspect to the existence of LPR.

The criteria for inclusion in the study were age 18 or older, subjects with histologically confirmed diagnosis of malignant or benign disease, and patients who operated septum deformation without symptoms and signs of LPR (RSI < 13, RFS < 7).

The criteria for exclusion from the study underwent therapies by proton pump inhibitors and/or antibiotics taken over the last month and RSI > 13 and RFS > 7 in control subjects.

All participants had taken a detailed history of the disease and a videolaryngoscopic examination. Prior to the operation, all patients had given saliva to determine the presence of pepsin due to protocol for saliva sampling and storage. All samples, 2 ml saliva spit through a straw into a testing tube, were taken in the morning before the first meal and brushing of teeth. Samples were transferred and stored in the refrigerator at 4°C while total pepsin concentration was measured within 2 days.

Laryngeal biopsy was performed in the general endotracheal anesthesia. All participants were introduced to the study purpose and protocol and signed informed consent approval for the examination procedure. The examination was conducted with the approval of the Ethical Committee of the University Hospital Centre Osijek and the Ethics Committee of the Faculty of Medicine in Osijek, in accordance with the Nuremberg Code and the latest revision of the Helsinki Declaration.

### Enzyme-linked immunosorbent assay

To determine the concentration of pepsin in saliva samples, we used the Enzyme-Linked Immunosorbent Assay (ELISA) method. This method is based on the application of antibodies and color change to determine concentrations of certain substances. A commercially available kit was used to determine pepsin: USCN LIFE SCIENCE Inc. Wuhan, China. It is also called Sandwich ELISA with a detection range of 3.12–200 ng/ml. At the end of the test, the absorbance at 450 nm on device 93200 PR3100 TSC Microplate Reader (Bio-Rad, U.S.A.) was measured. Using the standard samples, we drew a graphical representation. The absorbance value was shown at x, while the value of the pepsin concentration was on the y-axis. The final concentration of pepsin in saliva was read from the curve.

### Immunohistochemistry

Tissue sampled for immunohistochemistry analysis was changed laryngeal tissue: vocal polyps, vocal granulomas, mucosal membranes in Reinke’s edema, vocal nodules, laryngeal papillomatosis, *in situ* cancers, invasive carcinomas and mucosal interarytenoid membranes in the control subjects.

Expression of protein in tissue samples was followed by immunohistochemical (IHC) analysis of samples. Immunohistochemistry analysis was performed on permanent preparations fixed with 10% neutral formalin, incorporated in paraffin and cut into microtome, thickness of 3–5 micrometers. The cuts were deparaffined through a series of predicted solutions (xylene, 100, 96 and 70% ethanol). The samples were blocked by endogenous peroxidase with 0.3% hydrogen peroxide and then antigen retrieval was performed in the citrate buffer (pH 6.0) by heating in a microwave for ∼5 min. Primary antibody was applied to the preparations incubated at 4°C overnight, while PBS (pH 7.4) was applied to negative controls without primary antibody and were also held overnight under the same conditions. As a positive control, human stomach tissue was used. Positive controls passed the same procedure as the tested preparations. After incubation with the primary antibody, the cuts were washed in PBS in which Tween (0.05%) was added. Subsequently, the secondary antibody was applied to the preparations, which stayed at room temperature for 45 min. Next four rinses in PBS with 0.05% Tween followed. Then the incubation with Streptavidin-conjugated horseradish peroxidase (Streptavidin–HRP) was performed at room temperature for the next 45 min. After another four rinses in PBS with Tween, a solution of 3,3′-diaminobenzidine (DAB, substrate) was applied. After four rinses in PBS with Tween, the preparations were contrast painted with counterstaining and dehydrated in the solutions (70% ethanol, 96% ethanol and 100% ethanol and xylol). At the end of the procedure, the Canada balsam was applied to the preparation and covered with cover glass. Preparations were photographed with a digital camera connected to a microscope. QuickPHOTO Pro software was used for image editing and processing.

### Histological tissue analysis

Laryngeal tissue samples (vocal polyps, vocal granulomas, mucosa from REs, vocal nodules, laryngeal papillomatosis, *in situ* carcinomas, invasive carcinomas and mucosal membranes of the interarytenoid area in the control subjects) were removed and analyzed histomorphometrically.

The material for analysis was fixed in 10% neutral buffered formalin for 24 h at room temperature. After the fixation, the tissue was dehydrated in a series of alcohols from lower concentration to higher (70, 96, 100%). Then the alcohol was squeezed out from the tissue. Xylene was used for this purpose. Finally, the tissue was put in the impregnation medium, paraffin with melting point at 56–60°C. This part of tissue preparation was performed in the Histokinete tissue processor and lasted for ∼18 h. From the tissue processor, tissue bits were fitted into liquid paraffin. Molds were formed which were suitable for cutting blocks on a sliding microtome. The sections had a thickness of 3–5 microns. After that cover glass was stretched and mounted on the water bath. The cut and mounted preparations were dried in a thermostat overnight at a temperature of 56–60°C. After that they were ready for coloring. Before coloring, the preparations were deparaffined with xylene, then treated with alcohol (100, 96, 70%) and finally rinsed in water. For the coloring of the preparations for pathohistological diagnosis, Hemalaun—color for core, and Eosin—color for the cytoplasm, were used. After painting the preparations, they were rinsed in water and treated with an ascending range of alcohol (70, 96, 100%) and finally in xylene. The cover was put over the glass. All samples were analyzed by the same pathologist with the light microscope.

### Statistical analysis

Categorical data were represented by absolute and relative frequencies. Numerical data were described by the median and the limits of the interquartile range (IQR). Differences of categorical variables were tested by Fisher’s exact test. The normality of the distribution of numerical variables was tested by the Shapiro–Wilk test. The distribution of pepsin was normal. Differences between numerical variables were tested by Kruskal–Wallis test (post hoc Conover). The correlation between numerical variables was evaluated with Spearman’s correlation coefficient ρ (rho). All *P*-values are two-sided. The significance level was set to α = 0.05. The statistical analysis was performed using MedCalc Statistical Software version 19.4.1 (MedCalc Software Ltd, Ostend, Belgium; https://www.medcalc.org; 2020) and SPSS 17.0 (SPSS Inc., Chicago, IL, U.S.A.).

## Results

Of the 81 adult patients who participated in the study, the number of male patients was slightly higher, but significantly more in the group with malignant changes (Fisher’s exact test, *P*=0.001). By comparing the age of the participants in the three groups, a significant difference was found, patients with malignancies were significantly older than those with benign changes or from the control group (Kruskal–Wallis test). In the group with malignant lesions, the only diagnosis was PCC 25 (45%), while other diagnoses occurred in the group with benign lesions (Fisher’s exact test, *P*<0.001). In the group with malignant disease, 10 (40%) patients had stage T2 (Table 1).

**Table 1:** Characteristics of participants in observed groups

	Malignant	Benign	Control	Total	*P*
Gender [*n* (%)]					
Men	23 (92)	14 (45)	15 (60)	52 (64.2)	0.001*
Women	2 (8)	17 (55)	10 (40)	29 (35.8)	
Age [median (25–75%)]^‡^	58 (53–64)^‡^	48 (39–59)	47 (35–57)	52 (42–62)	0.002**^†^**
Diagnosis [*n* (%)]					
PCC	25 (100)	0	-	25 (45)	<0.001*
PAP	0	3 (10)	-	3 (5)	
VP	0	14 (45)	-	14 (25)	
RE	0	10 (32)	-	10 (18)	
Other	0	4 (13)	-	4 (7)	
Total	25 (100)	31 (100)		56 (100)	
T stage [*n* (%)]					
T1	7 (28)	-	-	-	-
T2	10 (40)	-	-	-	
T3	5 (20)	-	-	-	
T4	3 (12)	-	-	-	
Total	25 (100)	-	-	-	

* Fisher’s exact test.

† Kruskal–Wallis test (post hoc Conover).

‡ Level *P*<0.05 significant differences benign vs. malignant, benign vs. control.

Spearman’s correlation coefficient was used to assess the correlation between the age of the participants and the values of RSI, RFS and pepsin concentration in each group of participants. The only positive and significant correlation between RFS and age was observed in the group of patients with benign lesions (Rho = 0.595, *P*<0.001) (Table 2).

**Table 2:** Correlation between the age of the subjects and the values of RSI, RFS and pepsin concentration in each group of participants

	Spearman correlation coefficient Rho (*P*-value) participants age
Malignant	
RSI	0.001 (0.99)
RFS	0.031 (0.89)
Pepsin concentration	0.391 (0.09)
Benign	
RSI	0.010 (0.96)
RFS	0.595 (<0.001)
Pepsin concentration	0.024 (0.91)
Control group	
RSI	0.214 (0.30)
RFS	0.186 (0.37)
Pepsin concentration	0.083 (0.71)

Participants with malignant lesions had significantly higher values of RSI and RFS (Kruskal–Wallis test, *P*<0.001). RSI values differed significantly between participants in the control group and participants with malignant or benign lesions. While RFS showed significant differences between malignant and benign changes (Table 3).

**Table 3:** Correlation between each RSI and RFS symptom in three groups (malignant, benign and control)

	Malignant	Benign	Control	Total	*P*
^†^RSI [median (25–75%)]	12 (9–18)	9 (7–13)	2 (0–4)	8 (3–12)	<0.001^*^
^‡^RFS [median (25–75%)]	13 (11–17)	12 (9–14)	1 (0–3)	10 (3–13)	<0.001^*^

* Kruskal–Wallis test (post hoc conover).

† Level *P*<0.05 significant differences between benign and control, malignant and control.

‡ Level *P*<0.05 significant differences between benign and malignant, benign and control, malignant and control.

Significant difference between the three groups was found for RSI total, hoarseness, throat clearing, sensation of sticking in throat and heartburn. In all these cases, participants from the control group reported less on these indicators, they had a lower RSI (*P*<0.05). Significant differences are observed between the control group and the malignant or benign group.

When compared with the participants with benign lesions, in the group with malignant lesions, according to RFS, significantly more participants had complete and partial ventricular obliteration, diffuse hyperemia, moderate or severe diffuse laryngeal edema, adherent granuloma and severe posterior commissure hypertrophy. Severe vocal cord edema, polyp and thick laryngeal mucus were significantly more present in participants with benign lesions (Fisher’s exact test *P*<0.001) (Table 4).

**Table 4:** RSI and RFS in malignant, benign and control groups

	Malignant	Benign	Control	P*
**RSI** [median (25–75%)]				
Hoarseness	3 (3–4)	3 (2–4)	0 (0–1)	<0.001^†^
Clearing throat	2 (0–3)	3 (0–4)	0 (0–2)	0.003^†^
Excess throat mucus	0 (0–3)	0 (0–0)	0 (0–1)	0.08
Difficulty in swallowing	0 (0–1)	0 (0–1)	0 (0–0.5)	0.33
Coughing after meal of after lying down	0 (0–1.5)	0 (0–1)	0 (0–0)	0.06
Choking episodes	0 (0–0)	0 (0–0)	0 (0–0)	0.14
Annoying cough	0 (0–2.5)	0 (0–0)	0 (0–0)	0.08
Sensation of sticking in throat	2 (0–3)	2 (0–4)	0 (0–0.5)	<0.001 ^†^
Heartburn	1 (0–2)	2 (0–3)	0 (0–0)	<0.001 ^†^
**RFS** [*n* (%)]				
Subglottic edema	1 (4)	0	0	0.62^‡^
Ventricular obliteration				
Absent	2 (8)	11 (35)	25 (100)	<0.001^‡^
Partial	20 (80)	19 (61)	0	
Complete	3 (12)	1 (3)	0	
Hyperemia				
Absent	0	3 (10)	18 (72)	<0.001^‡^
Only arytenoids	3 (12)	13 (42)	6 (24)	
Diffuse	22 (88)	15 (48)	1 (4)	
Edema vocal fold				
Absent	0	0	23 (92)	<0.001^‡^
Mild	6 (24)	4 (13)	2 (8)	
Moderate	15 (60)	5 (16)	0	
Severe	4 (16)	6 (19)	0	
Polypoid	0	16 (52)	0	
Diffuse laryngeal edema				
Absent	7 (28)	21 (68)	25 (100)	<0.001^‡^
Mild	11 (44)	6 (19)	0	
Moderate	5 (20)	2 (6)	0	
Obstructive	2 (8)	2 (6)	0	
Posterior commissure hypertrophy				
Absent	1 (4)	0	13 (52)	<0.001^‡^
Mild	1 (4)	11 (35)	11 (44)	
Moderate	18 (72)	16 (52)	1 (4)	
Severe	4 (16)	4 (13)	0	
Obstructive	1 (4)	0 (0)	0	
Granuloma - present	25 (100)	9 (29)	0	<0.001^‡^
Thick endolaryngeal mucus - present	9 (36)	15 (48)	0	<0.001^‡^

* Kruskal–Wallis test (post hoc conover).

‡ Fisher’s exact test.

† Level *P*<0.05 significant differences benign and control, malignant and control.

## Concentration of pepsin in saliva

The highest concentration of pepsin in saliva was measured in the group with malignant lesions, followed by the group with benign changes, while the lowest concentration was measured in the control group. However, the differences are not statistically significant (Table 5).

**Table 5:** Difference in saliva pepsin concentration among three groups

	Malignant	Benign	Control	Total	*P*
Pepsin concentration [median (25–75%)]	200 (107–261)	175 (155–240)	152.5 (140–190)	169 (140.5–225)	0.16^*^

^*^Kruskal–Wallis test.

Although pepsin concentrations were slightly higher in participants diagnosed with PCC and RE, pepsin values did not differ significantly from the most common diagnoses (Table 6).

**Table 6:** Saliva pepsin concentration in the most common diagnoses

	*n*	Median (IQR) pepsin concentration	Minimum–maximum	P*
PCC	19	200 (106–265)	80–420	0.52
VP	14	172.5 (153.8–228.8)	99–295	
RE	6	232.5 (162.5–270)	155–300	

* Kruskal–Wallis test.

RSI and RFS do not correlate with saliva pepsin level either in malignant or benign group of patients, and there is no significant correlation of saliva pepsin level with any of the symptoms or clinical findings (Spearman’s correlation coefficient *P*>0.05), data not shown.

## Analysis of the results obtained by IHC method

**Figure 1:**
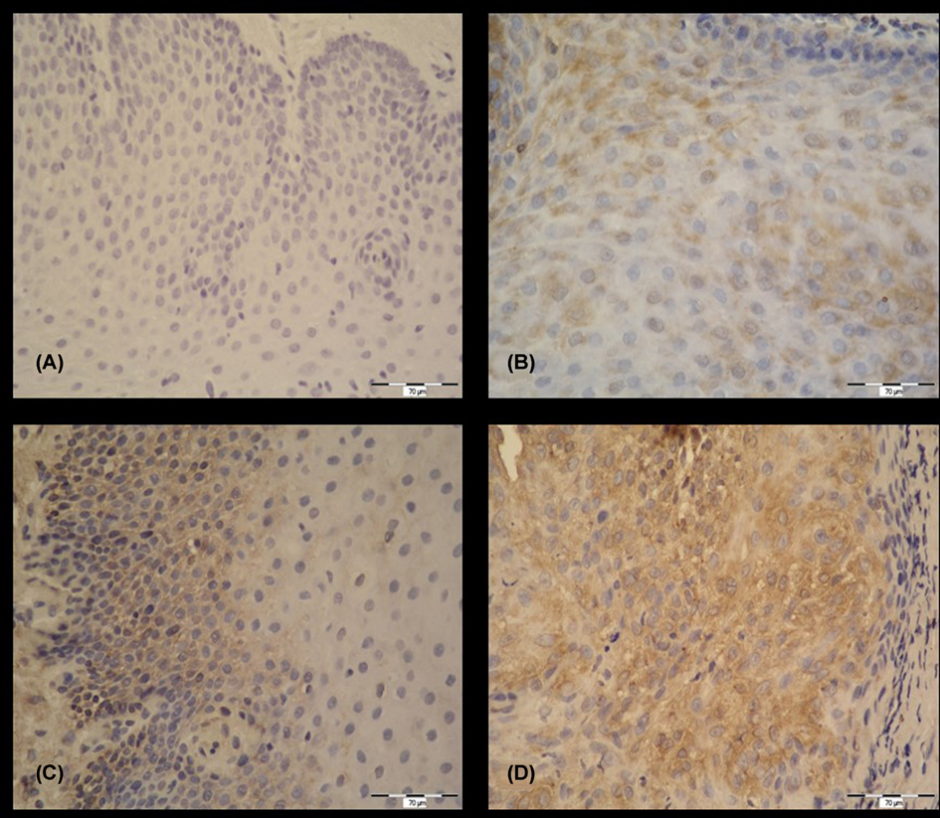
IHC detection of pepsin (magnification 400×) (**A**) Negative control, without IHC signal (−). (**B**) Healthy laryngeal tissue, IHC signal present in cytoplasm of epithelial cells, low positive (+). (**C**) IHC signal present in cytoplasm of epithelial cells, moderate positive (++). (**D**) IHC signal present in cell cytoplasm, high positive (+++).

Tissue samples of the interarytenoid area in the control subjects and tissue samples of benign and malignant lesions in the larynx were analyzed. IHC signal is seen in the form of brown intestinal cytoplasmic grains in the cytoplasm of epithelial cells. The intensity of the IHC signal was recorded in four categories as negative (−), low positive (+), moderate positive (++) and high positive (+++). For healthy volunteers, healthy gastric tissue was used (Figure 1).

IHC analysis showed significant intensity changes in IHC signal in significantly more subjects with malignant changes. The number of those who had high positive was significantly higher in the group with malignant lesions (Fisher’s exact test *P*<0.001). Moderate positive were observed significantly more frequently in benign lesions (including three laryngeal papillomatosis, two of them had high positive and the remaining were low positive). In the control group, none of the subjects had a high positive result (Table 7).

**Table 7:** Intensity of IHC signal in malignant and benign changes and control group

	Malignant changes	Benign changes	Control group	Total	*P*
Intensity IHC signal					
Negative	0	0	11 (58)	11 (16.9)	<0.001*
Low positive	5 (28)	8 (29)	6 (32)	19 (29.2)	
Moderate positive	4 (22)	15 (54)	2 (11)	21 (32.3)	
High positive	9 (50)	5 (18)	0	14 (21.5)	
Total	18 (100)	28 (100)	19 (100)	65 (100)	

* Fisher’s exact test.

The Kruskal–Wallis test showed a significant difference in the IHC signal to the concentration of pepsin in saliva between participants with malignant and benign lesions, and between malignant lesions and the control group. The intensity of the IHC signal is most pronounced in the group of malignant lesions and benign lesions with the highest level of pepsin concentration in saliva (Table 8).

**Table 8:** Correlation between intensity of IHC signal and pepsin concentration

	Median (25–75%) pepsin concentration and IHC								P*
	Negative	P*	Low positive	P*	Moderate positive	P*	High positive	P*	
Malignant changes (*n*=5:4:9)	-	-	89 (86–102,5)	0.04^†^	135 (108–175)	0.11	249 (209.5–305)	0.16	0.001^‡^
Benign changes (*n*=8:15:5)	-		125 (98–160)		180 (165–225)		300 (267.5–350)		<0.001^‡^
Control (*n*=11:6:2)	148 (105–162)		168 (140–205)		222.5 (220–225)		-		0.04^‡^

* Kruskal–Wallis test.

† Level *P*<0.05 significant differences between malignant vs. benign, malignant vs. control.

‡ Level *P*<0.05 significant differences between low positive and moderate positive, low positive and high positive, moderate positive and high positive.

We tried to establish whether higher results of RSI questionnaire can indicate increased intensity of IHC signal. The Kruskal–Wallis test showed no significant difference in RSI according to intensity of IHC signal and observed groups (Table 9).

**Table 9:** Correlation between intensity of IHC signal and RSI symptoms

	Median (25–75%) RSI and IHC								P*
	Negative	P*	Low positive	P*	Moderate positive	P*	High positive	P*	
Malignant (*n*=5:4:9)	-	-	9 (7.25–9.75)	0.005^†^	20 (11–26.5)	0.06	10 (9–12)	0.89	0.17
Benign (*n*=8:15:5)	-		9 (7.5–16.5)		9 (6.25–13.5)		10 (8.5–12.3)		0.86
Control (*n*=11:6:2)	3 (0.25–5)		1.5 (1–3)		1.5 (0–3)		-		0.74

* Kruskal–Wallis test.

† Level *P*<0.05 significant differences between malignant and control, benign and control.

The same was done for results of RFS questionnaire. There was no significant difference in RFS according to intensity of IHC signal and observed groups (Table 10).

**Table 10:** Correlation between intensity of IHC signal and RFS

	Median (25–75%) RSF and IHC								P*
	Negative	P*	Low positive	P*	Moderate positive	P*	High positive	P*	
Malignant (*n*=5:4:9)	-	-	13 (10–14.75)	0.002^†^	14 (10.5–19)	**0.04**^†^	13 (12.75–17.25)	0.05	0.69
Benign (*n*=8:15:5)	-		10 (9–12.5)		13 (9.25–14.75)		11 (9.25–13)		0.43
Control (*n*=11:6:2)	1 (0–1.75)		2.5 (1.5–3.5)		0 (0–0.5)		-		0.74

* Kruskal–Wallis test.

† Level *P*<0.05 significant differences between malignant and control, benign and control.

## Discussion

The result of the current study support the findings of other studies which state that LPR is a common occurence among patients with benign and malignant lesions. Our study established a higher mean level of total pepsin in the saliva in the group of patients with malignant lesions, followed by the group with benign lesions, while the lowest concentration was measured in the control group. However, differences were not statistically significant. The results indicated a higher concentration of pepsin in saliva in the group with benign and malignant changes in the larynx than in healthy individuals, which could be explained by a higher incidence of LPR in the two study groups. Some studies have demonstrated that pepsin detection in the saliva can be considered both as a sensitive and a non-invasive method for the diagnosis of LPR [16–19]. Pepsin is with bile acids and gastric acid part of gastric content which is produced in gastrointestinal tract below the level of larynx [20]. Results of our study indicate retrograde movement of pepsin, a part of gastric content, to the level of the larynx in malignant and benign changes groups as well as in the control group. These results correspond to the reserach in which pH metric measurements confirmed LPR in 60–100% patients with benign changes and 67–87% of malignant changes [21–25].

The scores of standardized Belafsky questionnaire, RSI and RFS, were also significantly higher in the group of malignant changes than in benign changes and control group. The present study did not show correlation between any of RSI and RFS symptoms and clinical finding with saliva pepsin concentration in any of three groups.

In recent literature, there are not many studies that used IHC analysis of laryngeal tissue samples to investigate concentration of pepsin. As demonstred, pepsin may be stored by an endocytosis receptor in the cell at an unfavorable pH value such as on the laryngeal mucosa [26]. It was expected that pepsin could be proved within the cells of the mucus membrane by the IHC method. Jiang et al. [27] analyzed samples of mucosal laryngeal tissue from the interarytenoid area to the presence of pepsin in two groups of subjects, those without symptoms of LPR and chronic laryngitis subjects. Their research has shown significant presence of pepsin in laryngeal mucosa cells in subjects with chronic laryngitis.

Our study showed all positive results in malignant and benign lesions in analyzed tissue samples. Only two samples from control group showed moderate positive result. Since Jiang et al. [27] IHC confirmed evidence of strong and moderate positivity in over 80% of tissue samples in LPR patients, it can be assumed that patients with benign and malignant changes have a high percentage of LPR. One of the most recent studies from 2016 also confirmed the hypothesis that expression of pepsin in laryngeal tissue is increased in patients with vocal leukoplakies and laryngeal carcinomas, contributing to the development of laryngeal carcinogenesis [28]. Results of IHC analysis suggest higher presence of pepsin in the laryngeal mucosal cells in benign and malignant diagnosis so it is more likely to damage the cells by direct action of pepsin within the cytoplasm of cells. Kelly et al. [29] have shown that chronic exposure of pepsin to *in vitro* oncogenic modified hypopharyngeal cells leads to their proliferation, migration and colony formation, thus giving additional importance to pepsin as an oncogenic transformation factor.

## Conclusion

Increased concentrations of pepsin in subjects with benign and malignant changes in the laryngeal mucosa indicate to LPR and pepsin as etiological factors in the formation of benign and malignant changes in the larynx. Concentrations of pepsin in saliva are higher in participants with a higher total sum of Belafsky questionnaires (RSIs, RFSs).

LPR is an important cofactor in the formation of benign and malignant changes of the larynx. Increased concentrations of pepsin in the saliva with the presence of characteristic signs and symptoms of LPR may indicate a high incidence of LPR in patients with benign and malignant changes in the larynx. Questionnaires are useful for raising doubts about the existence of laryngeal reflux in subjects with benign and malignant changes.

This research has contributed to the change in the present understanding of the etiopathogenesis of benign and malignant laryngeal diseases. Proper and timely diagnosis of LPR could change the preventive and therapeutic approach to benign and malignant diseases of the larynx.

## Abbreviations

CAIII: carboanhydrate III
ELISA: enzyme-linked immunosorbent assay
IHC: immunohistochemical
LPR: laryngopharyngeal reflux
PAP: laryngeal papillomatosis
PCC: planocellular carcinoma
RE: Reinke edema
VP: vocal cord polyp
